# Evaluating Checklist Use in Companion Animal Wellness Visits in a Veterinary Teaching Hospital: A Preliminary Study

**DOI:** 10.3389/fvets.2017.00087

**Published:** 2017-06-09

**Authors:** Michael T. Nappier, Virginia K. Corrigan, Lara E. Bartl-Wilson, Mark Freeman, Stephen Werre, Eric Tempel

**Affiliations:** ^1^Department of Small Animal Clinical Sciences, Virginia-Maryland College of Veterinary Medicine, Blacksburg, VA, United States; ^2^Laboratory for Study Design and Statistical Analysis, Virginia-Maryland College of Veterinary Medicine, Blacksburg, VA, United States

**Keywords:** checklists, wellness visits, Partners for Healthy Pets, companion animal, preventive health-care guidelines

## Abstract

The number of companion animal wellness visits in private practice has been decreasing, and one important factor cited is the lack of effective communication between veterinarians and pet owners regarding the importance of preventive care. Checklists have been widely used in many fields and are especially useful in areas where a complex task must be completed with multiple small steps, or when cognitive fatigue is evident. The use of checklists in veterinary medical education has not yet been thoroughly evaluated as a potential strategy to improve communication with pet owners regarding preventive care. The authors explored whether the use of a checklist based on the American Animal Hospital Association/American Veterinary Medical Association canine and feline preventive care guidelines would benefit senior veterinary students in accomplishing more complete canine and feline wellness visits. A group of students using provided checklists was compared to a control group of students who did not use checklists on the basis of their medical record notes from the visits. The students using the checklists were routinely more complete in several areas of a wellness visit vs. those who did not use the checklists. However, neither group of students routinely discussed follow-up care recommendations such as frequency or timing of follow-up visits. The study authors recommend considering checklist use for teaching and implementing wellness in companion animal primary care veterinary clinical teaching settings.

## Introduction

Wellness visits are an important part of routine veterinary care for dogs and cats. Despite that, the number of veterinary visits for both dogs and cats has been decreasing over the last several years (1). The Bayer Veterinary Care Usage study reported that up to 15% of dogs and 40% of cats had not been to a veterinarian in the previous year (2). The decrease in visits and gap in veterinary care for companion animals were attributed to several different factors. Key among these was an “inadequate understanding of the need for routine examinations.” (2) This lack of understanding was ascribed to clients associating the need for a veterinary visit only with vaccines and not with a need for other routine wellness care (3). In the Bayer study, 36% of owners felt that if their pet was not due for vaccines they would not bring them in for a veterinary visit, and 24% felt that routine wellness visits were unnecessary (2). These survey results highlight the disconnect in understanding of the value of routine wellness visits and preventative care between veterinary professionals and the pet owning public (2, 4, 5).

To help combat the decrease in veterinary visits and address the findings of the Bayer Veterinary Care Usage Study, the American Veterinary Medical Association (AVMA), the American Animal Hospital Association (AAHA), and other groups combined to form the Partners for Healthy Pets (PHP). The PHP provides resources for veterinarians to help identify specific communication gaps in their practice and better address them in a systematic manner. Among the items provided in their “toolkit” are the AAHA/AVMA canine and feline preventive health-care guidelines in the form of a checklist, which can be used during a wellness visit to help ensure all topics included in the preventive health guidelines are covered.

High-reliability organizations (HROs) such as aviation and nuclear fields have found checklists to be an effective way to reliably perform complex tasks in fields where higher levels of stress and fatigue are present. In particular, aviation has adapted checklist use to become a mandatory part of safe flight protocol (6, 7). Mandatory aviation checklist use can include a variety of normal activities. Introduction of newer electronic based checklists have increased efficiency even further (8, 9). In one example, implementation of the Boeing 777 Electronic Checklist decreased errors by 46% compared to traditional paper checklists (8). Use of checklists in human medicine has been more recent and has been less wide spread than other HROs. This may be partly due to a perception that medical tasks are too complex to be broken down into a simple checklist (10). Recently, several widely used human medical checklists have been developed and shown to be very effective (11–13). In 2008, the World Health Organization developed and implemented the Surgical Safety Checklist (SSC) to address common surgical errors. The SSC was shown to reduce mortality by 40% and surgical complications by 33% (14–17).

In human medical education, there have been several documented uses of checklists to improve retention of medical knowledge. Use of checklists during an internal medicine clerkship was found to increase exposure to procedures and improve acquisition of practical skills by at least 30% (18). In another case, checklists were used in a newborn medicine rotation and were found to improve transfer of information and student perception of their clinical experience (19). More recently, checklists were used at the Mayo Medical School for students in anatomic dissection laboratories. Students in an anatomy lab course were given daily checklists of objectives to accomplish. Checklist use was found to increase both student examination scores and dissection quality (20).

There has been very little written about the use of checklists for improving veterinary medical education. The lone available publication explored use of checklists with fourth year students for routine wellness visits and patient discharge visits following routine elective surgery (21). The study found that students using a checklist were routinely more thorough in communicating postoperative care instructions than students who did not use a checklist. Unfortunately, due to small sample size, no definitive conclusions were able to be made about checklist use for wellness visits.

The use of checklists in veterinary medical education has not yet been thoroughly evaluated as a potential strategy to improve communication with pet owners regarding preventive care. In this study, the authors explored whether the use of a checklist based on the AAHA/AVMA canine and feline preventive care guidelines would benefit senior veterinary students by helping them to accomplish systematic and complete canine and feline wellness visits.

## Materials and Methods

This study was performed through the small animal Community Practice service at the Veterinary Teaching Hospital of the Virginia-Maryland College of Veterinary Medicine (VMCVM). This study was carried out in accordance with the recommendations of the Virginia Tech Institutional Review Board, Protocol #16-109, with written informed consent from all subjects. All subjects gave written informed consent in accordance with the Declaration of Helsinki. The protocol was approved by the Virginia Tech Institutional Review Board.

**Figure 1:**
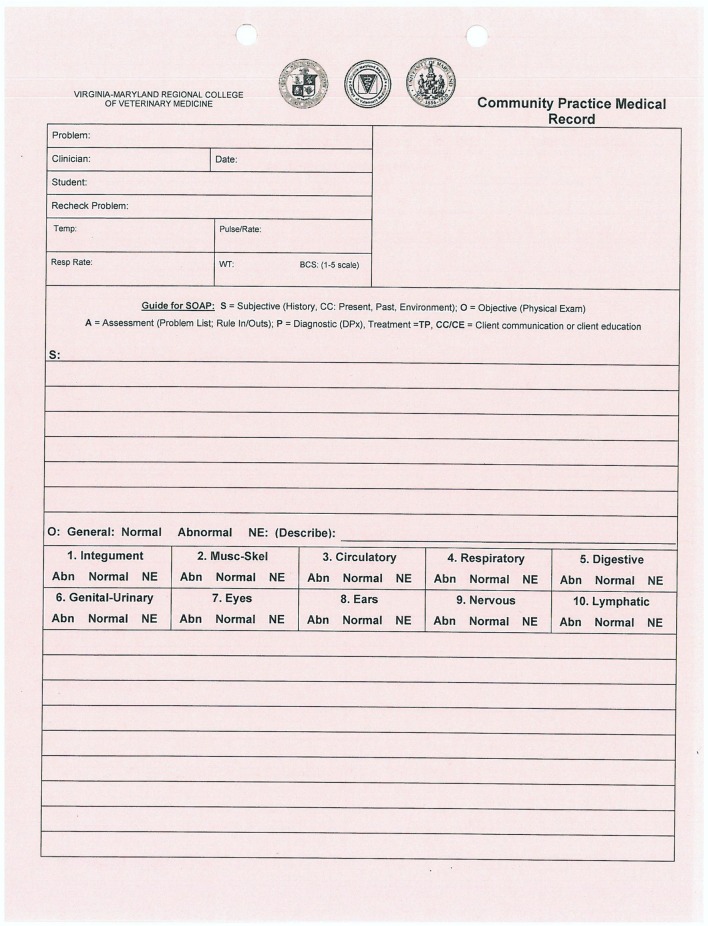
Example of student paper record form.

The students selected for participation in the study were fourth year veterinary students during their required 3-week clerkship in Community Practice over an 18-week period between May and September 2016. During the Community Practice clerkship at VMCVM, students are expected to see routine wellness appointments for both cats and dogs, including taking a patient history, performing a physical examination, and formulating a wellness health-care plan for the individual animal. Students normally record the history, exam findings, assessment, and plan on a paper record form designed for student use in working through the visit (Figure 1). After developing their assessment and plan, students present to the attending clinician, who suggests modifications as necessary.

**Figure 2:**
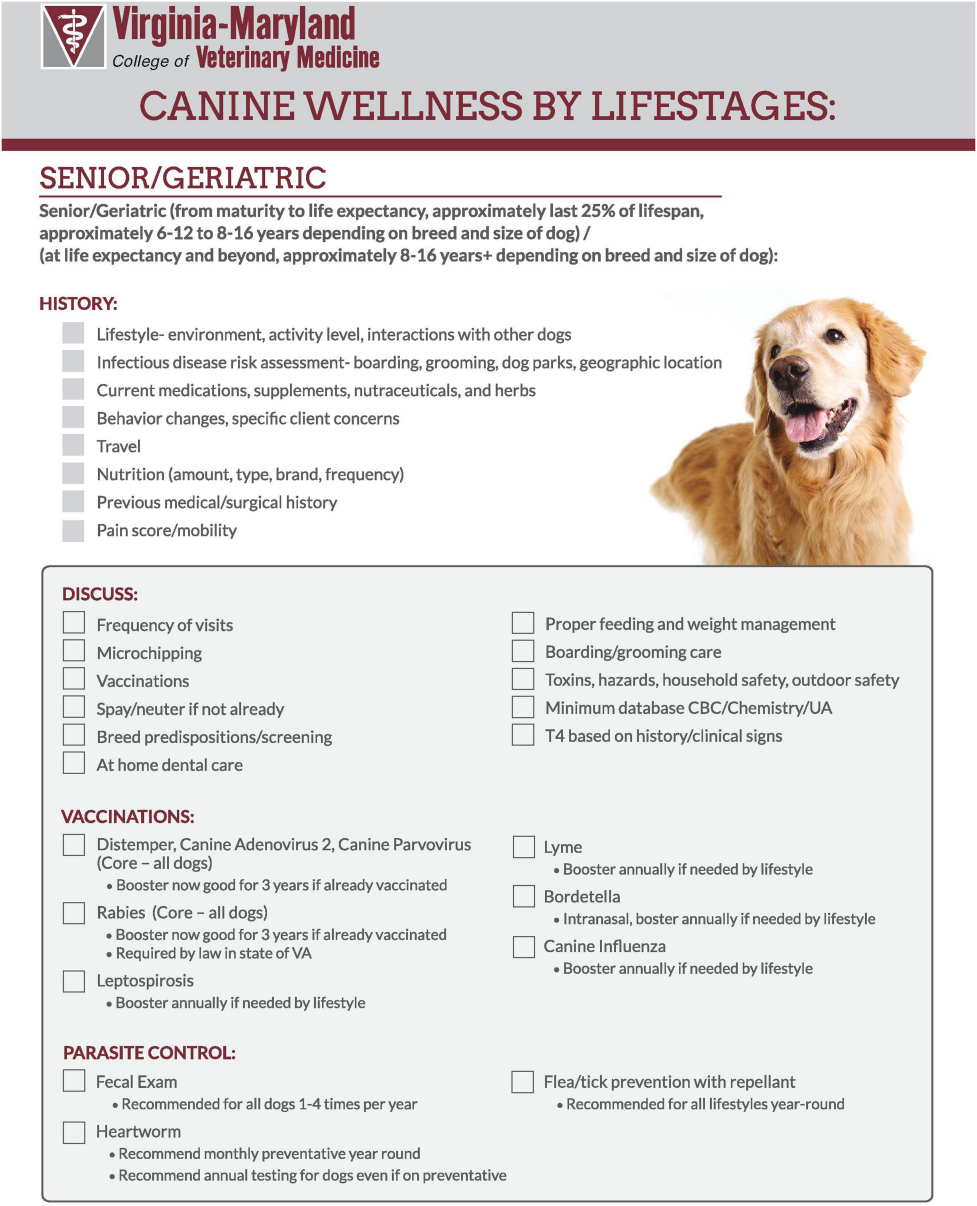
Example of modified checklist for senior dogs for Virginia-Maryland College of Veterinary Medicine.

Checklists for the VMCVM Community Practice were developed for wellness visits of all life stages for both cats and dogs. These checklists were based on the checklists available in the PHP toolkit. The checklists from PHP are meant to be universally usable for all areas of the country and as such contain some items which are either broadly stated or are inapplicable to the locale surrounding VMCVM. Using the AAHA/AVMA wellness guidelines for dogs and cats, the PHP checklists were altered to make specific recommendations tailored to the endemic risks in Southwest Virginia (Figure 2).

Students selected for the study were from the first six, 3-week clerkship blocks of the clinical year. Either seven or eight students were on the clerkship for each block. Consent for participation was obtained in accordance with Virginia Tech’s Institutional Review Board requirements. Fourteen students from the first three blocks who agreed to participate were used as the control group as they performed their wellness visits without the checklists. Nineteen students from the last three blocks who agreed to participate performed their wellness visits with the checklists. At the beginning of the block students were oriented to wellness visits with an hour-long seminar covering components of a complete wellness exam and introducing them to the contents of the PHP toolkit. After this seminar, the students in the last three blocks were given a set of checklists and instructed to use them for every wellness visit. For each wellness visit, students were asked to obtain a patient history, perform a physical examination, develop their assessment and wellness plan, and record it on the paper record form. Students were then asked to make a photocopy of the record form without any identifying information on it and place it in a collection box before consulting with their attending clinician. Puppy and kitten visits were excluded due to the inherently large variation of individual visit contents.

Nineteen major wellness topics listed on the checklists were organized into four groups for ease of analysis; history, vaccines, parasite control, and discussion topics. At the end of the study, copies of the record form were then analyzed by a single reviewer for both groups to determine if those topics were covered during the wellness visit.

Associations between use of checklists (check list used vs. check list not used) and each of the wellness topics (e.g., lifestyle/infectious risk: discussed vs. not discussed) were assessed using Fisher’s exact test. Statistical significance was set to *p* < 0.05. All analyses were performed using SAS version 9.4 (Cary, NC, USA).

## Results

A total of 95 wellness visits were analyzed (72 canine and 23 feline). Of those canine visits, 50 were without the checklists, and 22 were with the checklists. For the feline visits, 12 were without the checklists, and 11 were with the checklists. Results are presented in Table 1 (canine visits) and Table 2 (feline visits).

**Table 1:** Canine visit totals, percentages, and *p* values.

	Number without checklist	Number with checklist	% Without checklist	% With checklist	*p* Value
Total number	50	22			
History category total	50	22	100	100	N/A
Lifestyle/infectious disease risk	24	18	48	82	0.0094
Behavior	7	4	14	18	0.7264
Nutrition/current diet	36	17	72	77	0.7751
Previous medical history	40	21	80	95	0.1547
Current medications	20	15	40	68	0.0404
Pain/mobility	14	5	28	22	0.7751
Vaccinations category total	42	20	84	91	0.7131
Parasite control category total	45	22	90	100	0.3143
Heartworm test	25	19	50	86	0.0039
Fecal	7	9	14	41	0.0282
Flea/tick prevention	38	20	76	91	0.2012
Heartworm prevention	41	20	82	91	0.4847
Discussion topics category total	28	15	56	68	0.4362
Dental care	11	8	22	36	0.2498
Feeding/weight management recommendation	7	4	14		0.7264
Wellness labwork	9	2	18	9	0.4847
Breed predispositions	0	0	0	0	N/A
Frequency of visit/next visit	10	7	20	31	0.3673

**Table 2:** Feline visit totals, percentages, and *p* values.

	Number without checklist	Number with checklist	% Without checklist	% With checklist	*p* Value
Total number	12	11			
History category total	11	11	92	100	1.0000
Lifestyle/infectious disease risk	7	11	58	100	0.0373
Behavior	1	1	8	9	1.0000
Nutrition/current diet	9	9	75	82	1.0000
Previous medical history	11	11	92	100	1.0000
Current medications	1	7	8	64	0.0094
Pain/mobility	0	0	0	0	N/A
Vaccinations category total	11	11	92	100	1.0000
Parasite control category total	10	11	83	100	0.4783
FeLV/FIV status/test	5	6	42	55	0.6843
Fecal	2	3	17		0.6404
Flea/tick prevention	8	11	67	100	0.0932
Heartworm prevention	3	8	25	73	0.0391
Discussion topics category total	4	6	33	55	0.4136
Dental care	1	4	8	36	0.1550
Feeding/weight management recommendation	1	2	8	18	0.5901
Wellness labwork	2	0		0	0.4783
Breed predispositions	0	0	0	0	N/A
Frequency of visit/next visit	0	1	0	9	0.4783

Students were universally likely to discuss some part of three out of the four major visit areas (history, vaccinations, and parasite prevention). For dogs, students were quite likely to discuss prior medical problems, flea/tick prevention, and heartworm prevention. For cats, students were likely to discuss prior medical problems and current feeding practices. Students were generally unlikely to touch on any aspect of the discussion topics. This was especially unlikely for cats as only 33% of students without the checklists and 54% of students with the checklists remembered to discuss any of the topics.

Within the major visit areas, several areas of significant improvement were seen with students using the checklists vs. those without. For dogs, students using the checklists were significantly more likely to have discussed lifestyle/infectious disease risk (*p* = 0.009), current medications (*p* = 0.04), heartworm testing (*p* = 0.004), and fecal parasite testing (*p* = 0.0028). For cats, students with the checklists were significantly more likely to have discussed lifestyle/infectious disease risk (*p* = 0.037), current medications (*p* = 0.009), and heartworm prevention (*p* = 0.039).

## Discussion

This study builds on the known effectiveness of checklists and further expands their use into veterinary medicine and veterinary medical education. In particular, the checklists developed by PHP from the AAHA/AVMA guidelines were shown to be effective in helping students perform a more complete wellness visit. However, the checklists were not found to be universally effective and did not appear to prompt students to be more likely to discuss ongoing health management such as dental care or timing/frequency of next visit.

Checklists are beginning to become important and effective teaching tools in medicine. In this study, it was shown that while students generally did well at remembering to cover the broader topics of a wellness visit, many of the more specific details were often omitted. Since checklists are known to improve reliability for complex tasks in high stress and high fatigue instances, the fact that checklist use was shown to improve student completion of wellness visits in and of itself is not surprising. What was most useful and interesting from a teaching perspective were the specific discussion topics students had difficulty remembering and the fact that even with the checklists, ongoing preventive health management topics were still frequently missed.

There were several limitations to the current study. Students using the checklist were more thorough than those who did not use the checklists. However, the study did not investigate whether mandatory checklist use improved students’ retention of the topics that should be covered in a wellness visit, or exactly how the checklists were being utilized in the examination room.

Although the investigators were unable to identify any individual students, as no identifying information was recorded on the form submitted, investigators were not blinded when analyzing the forms and were aware of whether or not the student was in the group that used checklists. This has the potential to introduce observer bias in the analysis of the student forms.

An unintended consequence of the need for student confidentiality was that the investigators were unable to analyze the data by student and therefore unable to perform a multilevel analysis. Another unintended consequence was that while the total numbers of students who participated both with and without checklists is known, no further data regarding individual students were able to be analyzed, which may have erased the ability to control for confounding variables. For example, the investigators were unable to include the number of patients an individual student saw during the course of the block.

While it was the investigators’ judgment that all six blocks of students were of a similar ability and confidence level, the use of the checklists was not randomized. This could potentially introduce selection bias, as the first three blocks of students could have a significantly different ability level than the second three blocks.

The study also did not evaluate the students’ or clients’ perception of the use of checklists. While instructor mandated use of checklists improved student outcome in completeness of visits, it is not known if the students perceived the checklists to be easy to use and would therefore continue to use them voluntarily when performing wellness visits. Checklists that are viewed unfavorably by the user would stand little chance of being used long term; therefore, any benefit found in their use would be negated by a lack of compliance in use. Investigation of client perception would also potentially be worthwhile, as it is possible that veterinarians seen using checklists in the exam room would be perceived as unknowledgeable instead of thorough, thereby negating their benefit. Additionally, it would be of benefit to determine the level of understanding and/or retention by the pet owners of the information discussed during the visits with and without the checklists. Another interesting avenue of potential further investigation would be to determine if the students who used checklists were better able to translate the ability to accomplish a systematic and complete wellness exam in clinical practice after graduation.

The study data suggest that discussion of ongoing preventive health topics did not happen frequently either with or without the checklists. It has been previously shown that veterinarians in general are not effective at communicating the benefit of ongoing preventive care, and it is possible this is being reflected in the findings (4, 5). However, it is also plausible that the design of the study resulted in an artificially low reflection of how often these topics were being discussed. Students were asked to make the copy of their record form after taking a patient history, performing a physical examination, and developing a wellness plan, but before presenting to the attending veterinarian. The objective was to determine only what the student was able to do on their own without any help from the attending veterinarian. However, chronologically, submission of the record occurred approximately half way through the average visit. Since many of the preventive care recommendations and discussions happen toward the end of a visit, it is possible that students did come up with and discuss more preventive care recommendations but did not record them for the study as their record forms had already been submitted. It is possible that for further investigation, a complete audio recording of the visit would be useful to determine if more preventive care discussions actually happened than this study reports.

Extrapolation of the study results may provide evidence to recommend use in the checklist’s original application: companion animal private practice. It could be posited that the same sort of conditions that veterinary students experience (complex task, high stress, and high fatigue) also exist in a small animal private practice. It is likely that the exact areas of benefit would likely differ between student and practitioner/technician use, as well as individually between different private practices.

## Conclusion

The results of this preliminary study suggest that a modified version of the PHP wellness checklist based on the AAHA/AVMA preventive health-care guidelines may be an effective tool for teaching veterinary students to be able to perform systematic and complete companion animal wellness exams. Future prospective studies could be designed to assess implications of checklist use, including whether checklists lead to better communication with pet owners regarding preventive care, and thereby have the potential to improve the decline of wellness visits in companion animal practice. The study authors recommend checklist use for teaching and implementing wellness in companion animal primary care veterinary clinical teaching settings.

## Ethics Statement

This study was carried out in accordance with the recommendations of the Virginia Tech Institutional Review Board, Protocol #16-109, with written informed consent from all subjects. All subjects gave written informed consent in accordance with the Declaration of Helsinki. The protocol was approved by the Virginia Tech Institutional Review Board.

## Author Contributions

All the authors (MN, VC, LB-W, MF, SW, and ET) have provided the following contributions to the manuscript: (1) substantial contributions to the conception or design of the work; or the acquisition, analysis, or interpretation of data for the work; (2) drafting the work or revising it critically for important intellectual content; (3) final approval of the version to be published; and (4) agreement to be accountable for all aspects of the work in ensuring that questions related to the accuracy or integrity of any part of the work are appropriately investigated and resolved.

## Conflict of Interest Statement

This research was conducted in the absence of any commercial or financial relationships that could be construed as a potential conflict of interest.
